# Supplementary material for: What proportion of people have a follow-up biopsy in randomized trials of treatments for non-alcoholic steatohepatitis?: A systematic review and meta-analysis
Source: PLoS One. 2021 Apr 21;16(4):e0250385. doi: 10.1371/journal.pone.0250385 (PMC8059856; doi:10.1371/journal.pone.0250385)
Supplement: S1 File — (PDF) [file pone.0250385.s002.pdf]

S1 File

What proportion of people have a follow-up biopsy in randomised trials of treatments for non-alcoholic steatohepatitis?

Dimitrios Koutoukidis, Elizabeth Morris, John A Henry, Yusra Shammoon, Matthew Zimmerman, Moscho Michalopoulou, Susan A Jebb, Paul Aveyard

S1 Table. Characteristics of included studies

Study	Description of interventions	Time until first follow-up biopsy	Time until second follow-up biopsy
Chalasani 2020, USA [1]	Belapectin 2mg vs. belapectin 8mg vs. placebo	1 year	-
Abdelmalek 2009, USA [2]	Betaine vs. placebo	1 year	-
Friedman 2018, Multiple [3, 4]	Cenicriviroc vs. placebo vs. placebo for year 1 and ceinciviroc vs. ceinciviroc vs. placebo for year 2	1 year	2 years
Ratziu 2016, Multiple [5]	Elafibranor 80mg vs. elafibranor 120mg vs. placebo	1 year	-
Harrison 2020b, Multiple [6]	Emricasan 50mg vs. emricasan 5mg vs. placebo	1.4 years	-
Sanyal 2014, USA [7]	Ethyl-Eicosapentanoic Acid 1800mg vs. ethyl-eicosapentanoic acid 2700mg vs. placebo	1 year	-
Idilman 2008, Turkey [8]	Insulin sensitizers (either metformin or rosiglitazone) with diet and exercise vs. diet and exercise	1 year	-
Armstrong 2016, UK [9]	Liraglutide vs. placebo	1 year	-
McPherson 2017, UK [10]	Losartan vs. placebo	1.8 years	-
Sturm 2009, France [11]	Metformin, pentoxifylline, diet, and exercise vs. diet and exercise	1 year	-
Shields 2009, USA [12]	Metformin vs. placebo	1 year	-
Omer 2010, Turkey [13]	Metformin vs. rosiglitazone vs. metformin and rosiglitazone	1 year	-
Harrison 2020c, USA [14]	MSDC-0602K 62.5mg vs. MSDC-0602K 125mg vs. MSDC-0602K 250mg vs. placebo	1 year	-
Dasarathy 2015, USA [15]	n-3 fatty acids vs. placebo	1 year	-
Argo 2015, USA [16]	n-3 fatty acids with diet and exercise vs. placebo with diet and exercise	1 year	-
Oliveira 2019, Brazil [17]	n-acetylcysteine and ursodeoxycholic acid and metformin vs. ursodeoxycholic acid and metformin vs. n-acetylcysteine and metformin	1 year	-
Younossi 2019, Multiple [18]	Obeticholic acid 10mg vs. obeticholic acid 25mg vs. placebo	1.5 years	-
Neuschwander-Tetri 2015, USA [19]	Obeticholic acid vs. placebo	1.4 years	-
Van Wagner 2011, USA [20]	Pentoxifylline vs. placebo	1 year	-
Zein 2011, USA [21]	Pentoxifylline vs. placebo	1 year	-
Alam 2017, Bangladesh [22]	Pentoxifylline with diet and exercise vs. diet and exercise	1 year	-

Study	Description of interventions	Time until first follow-up biopsy	Time until second follow-up biopsy
Cusi 2016, USA [23]	Pioglitazone for 36 months vs. placebo then pioglitazone for 18 months	1.5 years	3 years
Aithal 2008, UK [24]	Pioglitazone vs. placebo	1 year	-
Ratziu 2008, France [25]	Rosiglitazone vs. placebo	1 year	2 years
Torres 2011, USA [26]	Rosiglitazone vs. rosiglitazone and metformin vs. rosiglitazone and losartan	1 year	-
Harrison 2020a - STELLAR 3, Multiple [27]	Selonsertib 18mg vs. selonsertib 6mg vs. placebo	1 year	-
Harrison 2020a- STELLAR 4, Multiple [27]	Selonsertib 18mg vs. selonsertib 6mg vs. placebo	1 year	-
Navarro 2019, USA [28]	Silymarin 420mg vs. silymarin 700mg vs. placebo	1 year	-
Wah Kheong 2017, Malaysia [29]	Silymarin vs. placebo	1 year	-
Nelson 2009, USA [30]	Simvastatin vs. placebo	1 year	-
Alam 2018, Bangladesh [31]	Sitagliptin with diet and exercise vs. diet and exercise	1 year	-
Leuschner 2010, Germany, Greece [32]	Ursodeoxycholic acid vs. placebo	1.5 years	-
Lindor 2004, USA, Canada [33]	Ursodeoxycholic acid vs. placebo	2 years	-
Dufour 2006, Switzerland [34]	Ursodeoxycholic Acid with vitamin E vs. ursodeoxycholic acid with placebo vs. two placebos	2 years	-
Georgescu 2009, Romania [35]	Valsartan vs. telmisartan	1.7 years	-
Geier 2018, Switzerland [36]	Vitamin D vs. placebo	1 year	-
Bril 2019, USA [37]	Vitamin E and placebo vs. pioglitazone and placebo vs. two placebos	1.5 years	-
Sanyal 2010, USA [38]	Vitamin E vs. pioglitazone vs. placebo	1.8 years	-
Alam 2020, Bangladesh [39]	Vitamin E with diet and exercise vs. telmisartan with diet and exercise	1 year	-
Newsome 2020, USA, Canada, UK [40]	Volixibat 5mg vs. volixibat 10mg vs. volixibat 20mg vs. placebo	1 year	-
Promrat 2010, USA [41]	Weight loss programme of diet and exercise vs. basic advice on diet and exercise	1 year	-

S1 Fig. Participant-related reasons for lacking a valid follow-up biopsy reported for N per 1,000 participants (95% CI).

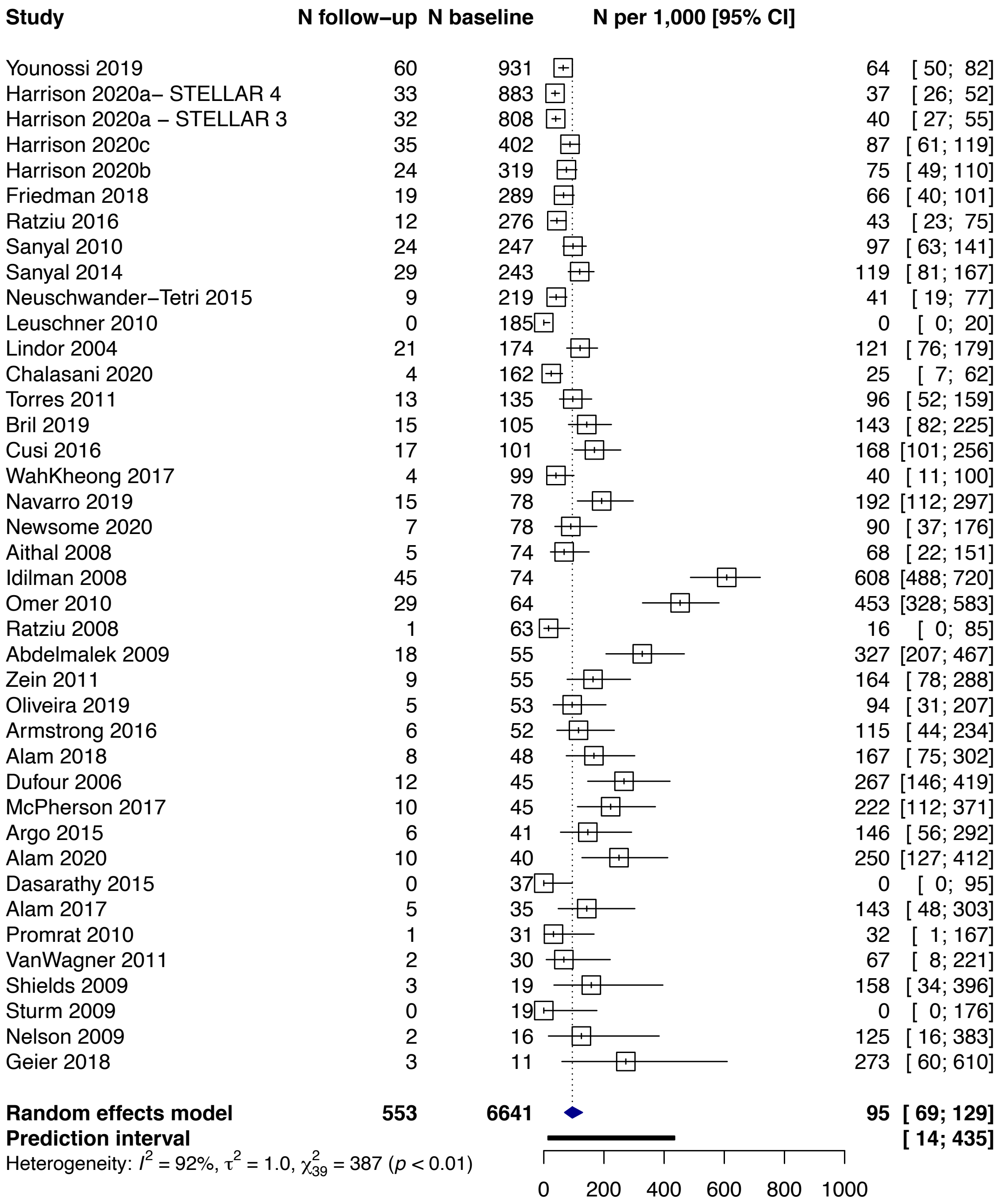

S2 Fig. Medically-related reasons for lacking a valid follow-up biopsy reported for N per 1,000 participants (95% CI).

Study	N follow–up	N baseline	N per 1,000 [95% CI]
Younossi 2019	0	931	0 [0; 4]
Harrison 2020a– STELLAR 4	40	883	45 [33; 61]
Harrison 2020a – STELLAR 3	18	808	22 [13; 35]
Harrison 2020c	5	402	12 [4; 29]
Harrison 2020b	7	319	22 [9; 45]
Friedman 2018	8	289	28 [12; 54]
Ratziu 2016	19	276	69 [42; 105]
Sanyal 2010	1	247	4 [0; 22]
Sanyal 2014	13	243	53 [29; 90]
Neuschwander–Tetri 2015	2	219	9 [1; 33]
Leuschner 2010	0	185	0 [0; 20]
Lindor 2004	23	174	132 [86; 192]
Chalasani 2020	6	162	37 [14; 79]
Torres 2011	12	135	89 [47; 150]
Bril 2019	6	105	57 [21; 120]
Cusi 2016	1	101	10 [0; 54]
WahKheong 2017	3	99	30 [6; 86]
Navarro 2019	0	78	0 [0; 46]
Newsome 2020	22	78	282 [186; 395]
Aithal 2008	6	74	81 [30; 168]
Idilman 2008	0	74	0 [0; 49]
Omer 2010	0	64	0 [0; 56]
Ratziu 2008	1	63	16 [0; 85]
Abdelmalek 2009	0	55	0 [0; 65]
Zein 2011	0	55	0 [0; 65]
Oliveira 2019	10	53	189 [94; 320]
Armstrong 2016	0	52	0 [0; 68]
Alam 2018	0	48	0 [0; 74]
Dufour 2006	0	45	0 [0; 79]
McPherson 2017	1	45	22 [1; 118]
Argo 2015	1	41	24 [1; 129]
Alam 2020	0	40	0 [0; 88]
Dasarathy 2015	0	37	0 [0; 95]
Alam 2017	0	35	0 [0; 100]
Promrat 2010	1	31	32 [1; 167]
VanWagner 2011	2	30	67 [8; 221]
Shields 2009	0	19	0 [0; 176]
Sturm 2009	2	19	105 [13; 331]
Nelson 2009	0	16	0 [0; 206]
Geier 2018	0	11	0 [0; 285]
**Random effects model**	**210**	**6641**	**16 [8; 29]**
**Prediction interval**			**[1; 246]**

Heterogeneity: $I^2 = 91\%$, $\tau^2 = 2.3$, $\chi^2_{39} = 158$ ($p < 0.01$)

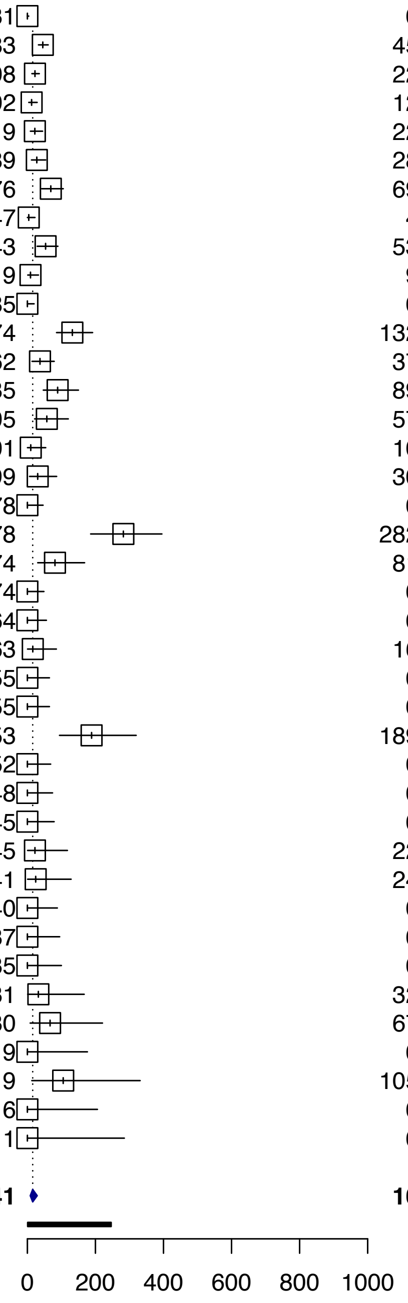

S3 Fig. Protocol-related reasons for lacking a valid follow-up biopsy reported for N per 1,000 participants (95% CI).

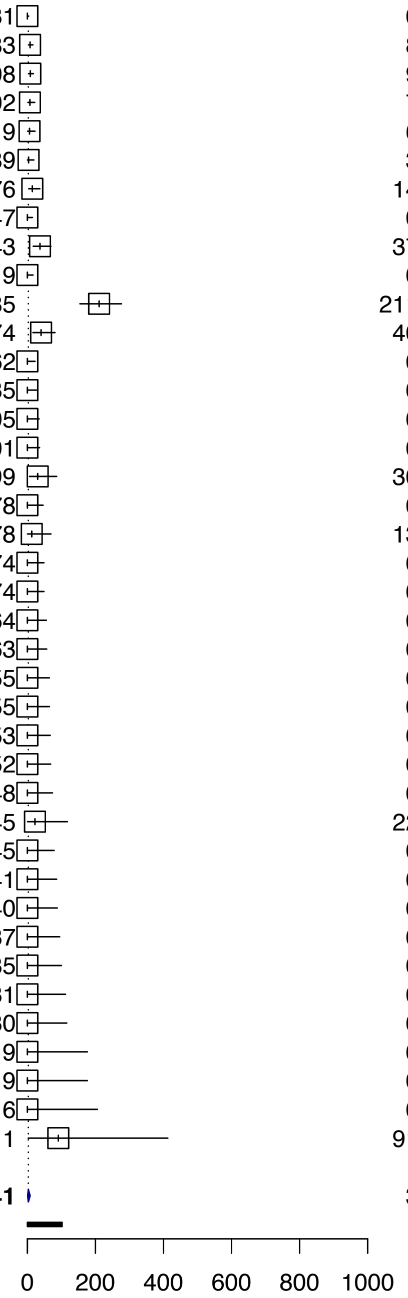

Study	N follow−up	N baseline	N per 1,000 [95% CI]
Younossi 2019	0	931	0 [0; 4]
Harrison 2020a− STELLAR 4	7	883	8 [3; 16]
Harrison 2020a − STELLAR 3	7	808	9 [3; 18]
Harrison 2020c	3	402	7 [2; 22]
Harrison 2020b	2	319	6 [1; 22]
Friedman 2018	1	289	3 [0; 19]
Ratziu 2016	4	276	14 [4; 37]
Sanyal 2010	0	247	0 [0; 15]
Sanyal 2014	9	243	37 [17; 69]
Neuschwander−Tetri 2015	0	219	0 [0; 17]
Leuschner 2010	39	185	211 [154; 277]
Lindor 2004	7	174	40 [16; 81]
Chalasani 2020	0	162	0 [0; 23]
Torres 2011	0	135	0 [0; 27]
Bril 2019	0	105	0 [0; 35]
Cusi 2016	0	101	0 [0; 36]
WahKheong 2017	3	99	30 [6; 86]
Navarro 2019	0	78	0 [0; 46]
Newsome 2020	1	78	13 [0; 69]
Aithal 2008	0	74	0 [0; 49]
Idilman 2008	0	74	0 [0; 49]
Omer 2010	0	64	0 [0; 56]
Ratziu 2008	0	63	0 [0; 57]
Abdelmalek 2009	0	55	0 [0; 65]
Zein 2011	0	55	0 [0; 65]
Oliveira 2019	0	53	0 [0; 67]
Armstrong 2016	0	52	0 [0; 68]
Alam 2018	0	48	0 [0; 74]
Dufour 2006	1	45	22 [1; 118]
McPherson 2017	0	45	0 [0; 79]
Argo 2015	0	41	0 [0; 86]
Alam 2020	0	40	0 [0; 88]
Dasarathy 2015	0	37	0 [0; 95]
Alam 2017	0	35	0 [0; 100]
Promrat 2010	0	31	0 [0; 112]
VanWagner 2011	0	30	0 [0; 116]
Shields 2009	0	19	0 [0; 176]
Sturm 2009	0	19	0 [0; 176]
Nelson 2009	0	16	0 [0; 206]
Geier 2018	1	11	91 [2; 413]
**Random effects model**	**85**	**6641**	**3 [1; 8]**
**Prediction interval**			**[0; 102]**

Heterogeneity: $I^2 = 86\%$, $\tau^2 = 3.3$, $\chi^2_{39} = 167$ ($p < 0.01$)

S4 Fig. Trial conduct-related reasons for lacking a valid follow-up biopsy reported for N per 1,000 participants (95% CI).

Study	N follow-up	N baseline	N per 1,000 [95% CI]
Younossi 2019	0	931	0 [0; 4]
Harrison 2020a– STELLAR 4	0	883	0 [0; 4]
Harrison 2020a – STELLAR 3	0	808	0 [0; 5]
Harrison 2020c	29	402	72 [49; 102]
Harrison 2020b	0	319	0 [0; 11]
Friedman 2018	8	289	28 [12; 54]
Ratziu 2016	0	276	0 [0; 13]
Sanyal 2010	0	247	0 [0; 15]
Sanyal 2014	0	243	0 [0; 15]
Neuschwander–Tetri 2015	0	219	0 [0; 17]
Leuschner 2010	0	185	0 [0; 20]
Lindor 2004	16	174	92 [53; 145]
Chalasani 2020	0	162	0 [0; 23]
Torres 2011	0	135	0 [0; 27]
Bril 2019	0	105	0 [0; 35]
Cusi 2016	0	101	0 [0; 36]
WahKheong 2017	0	99	0 [0; 37]
Navarro 2019	0	78	0 [0; 46]
Newsome 2020	0	78	0 [0; 46]
Aithal 2008	2	74	27 [3; 94]
Idilman 2008	0	74	0 [0; 49]
Omer 2010	0	64	0 [0; 56]
Ratziu 2008	0	63	0 [0; 57]
Abdelmalek 2009	2	55	36 [4; 125]
Zein 2011	0	55	0 [0; 65]
Oliveira 2019	0	53	0 [0; 67]
Armstrong 2016	1	52	19 [0; 103]
Alam 2018	0	48	0 [0; 74]
Dufour 2006	0	45	0 [0; 79]
McPherson 2017	2	45	44 [5; 151]
Argo 2015	1	41	24 [1; 129]
Alam 2020	0	40	0 [0; 88]
Dasarathy 2015	1	37	27 [1; 142]
Alam 2017	0	35	0 [0; 100]
Promrat 2010	1	31	32 [1; 167]
VanWagner 2011	1	30	33 [1; 172]
Shields 2009	0	19	0 [0; 176]
Sturm 2009	0	19	0 [0; 176]
Nelson 2009	0	16	0 [0; 206]
Geier 2018	0	11	0 [0; 285]
**Random effects model**	**64**	**6641**	**1 [0; 5]**
**Prediction interval**			**[0; 179]**

Heterogeneity: $I^2 = 91\%$, $\tau^2 = 6.8$, $\chi^2_{39} = 15$ ($p = 1.00$)

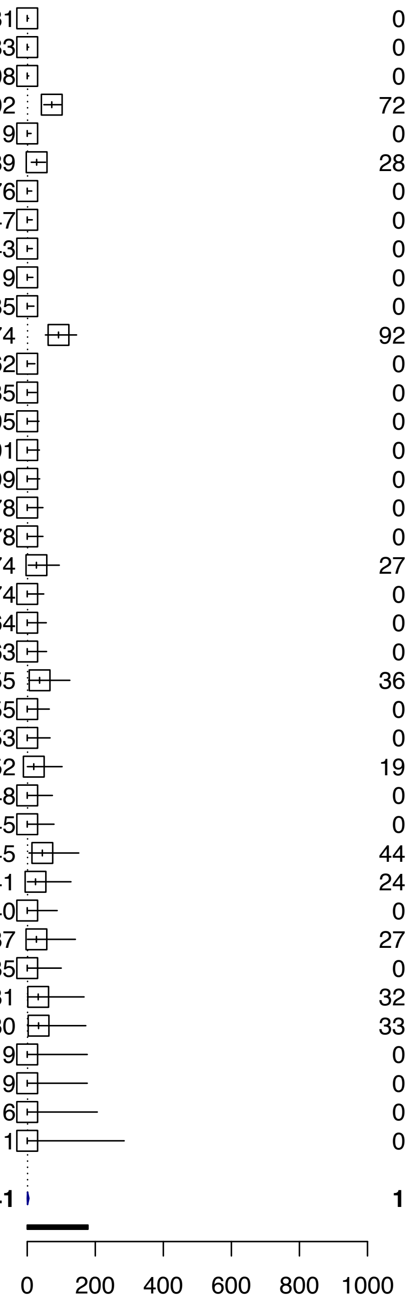

S5 Fig. Other/unclear reasons for lacking a valid follow-up biopsy reported for N per 1,000 participants (95% CI).

Study	N follow-up	N baseline	N per 1,000 [95% CI]
Younossi 2019	93	931	100 [81; 121]
Harrison 2020a– STELLAR 4	11	883	12 [6; 22]
Harrison 2020a – STELLAR 3	0	808	0 [0; 5]
Harrison 2020c	4	402	10 [3; 25]
Harrison 2020b	0	319	0 [0; 11]
Friedman 2018	1	289	3 [0; 19]
Ratziu 2016	4	276	14 [4; 37]
Sanyal 2010	0	247	0 [0; 15]
Sanyal 2014	18	243	74 [44; 115]
Neuschwander–Tetri 2015	8	219	37 [16; 71]
Leuschner 2010	7	185	38 [15; 76]
Lindor 2004	0	174	0 [0; 21]
Chalasani 2020	1	162	6 [0; 34]
Torres 2011	2	135	15 [2; 52]
Bril 2019	0	105	0 [0; 35]
Cusi 2016	0	101	0 [0; 36]
WahKheong 2017	0	99	0 [0; 37]
Navarro 2019	1	78	13 [0; 69]
Newsome 2020	5	78	64 [21; 143]
Aithal 2008	0	74	0 [0; 49]
Idilman 2008	0	74	0 [0; 49]
Omer 2010	0	64	0 [0; 56]
Ratziu 2008	0	63	0 [0; 57]
Abdelmalek 2009	0	55	0 [0; 65]
Zein 2011	0	55	0 [0; 65]
Oliveira 2019	0	53	0 [0; 67]
Armstrong 2016	0	52	0 [0; 68]
Alam 2018	0	48	0 [0; 74]
Dufour 2006	0	45	0 [0; 79]
McPherson 2017	0	45	0 [0; 79]
Argo 2015	0	41	0 [0; 86]
Alam 2020	0	40	0 [0; 88]
Dasarathy 2015	0	37	0 [0; 95]
Alam 2017	0	35	0 [0; 100]
Promrat 2010	0	31	0 [0; 112]
VanWagner 2011	0	30	0 [0; 116]
Shields 2009	0	19	0 [0; 176]
Sturm 2009	5	19	263 [91; 512]
Nelson 2009	4	16	250 [73; 524]
Geier 2018	0	11	0 [0; 285]
**Random effects model**	**164**	**6641**	**3 [1; 10]**
**Prediction interval**			**[0; 243]**

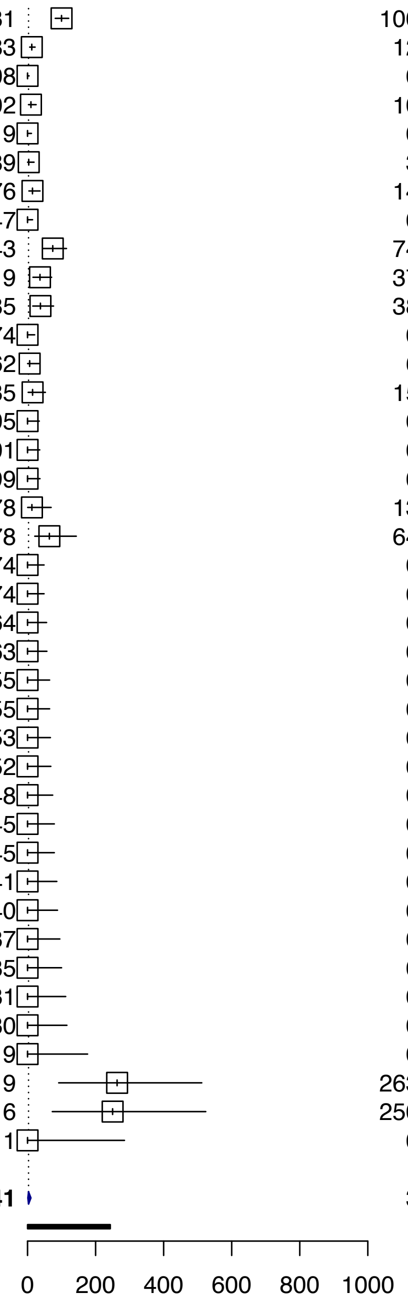

Heterogeneity: $I^2 = 94\%$, $\tau^2 = 5.3$, $\chi^2_{39} = 119$ ($p < 0.01$)

S6 Fig. Proportion of participants (95% CI) with a valid follow-up biopsy in trials conducted in high- and middle-income counties.

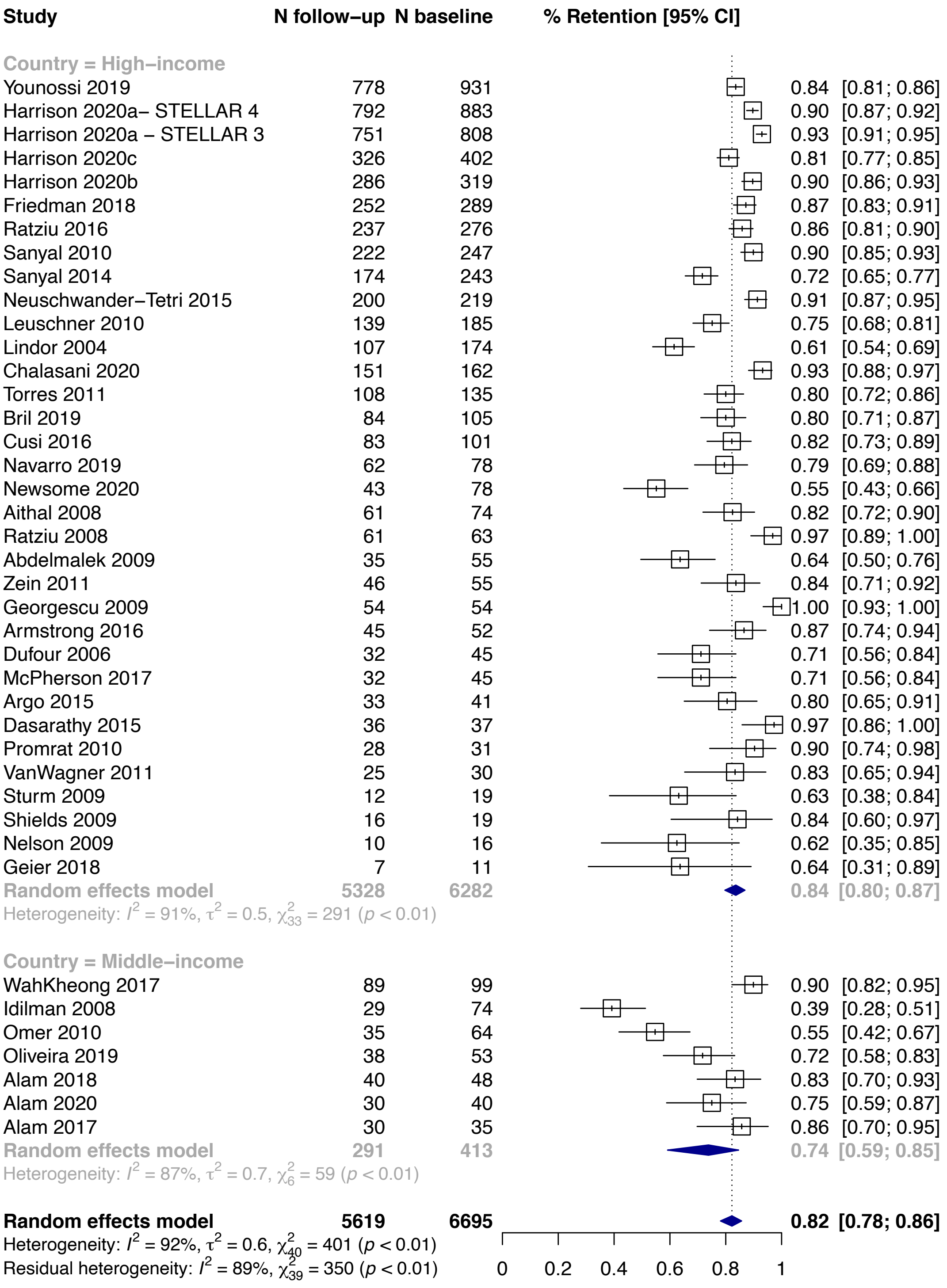

Test for subgroup differences (random effects model): p-value=0.11.
Following feedback from peer-reviewers, we also conducted a meta-regression by location. This confirmed the lack of evidence of a difference in the proportion of follow-up biopsies in middle-income compared with high-income countries [b = -0.60 (95% CI: -1.26 to 0.05, p=0.07)].

S7 Fig. Proportion of participants (95% CI) with a valid follow-up biopsy by geographical region.

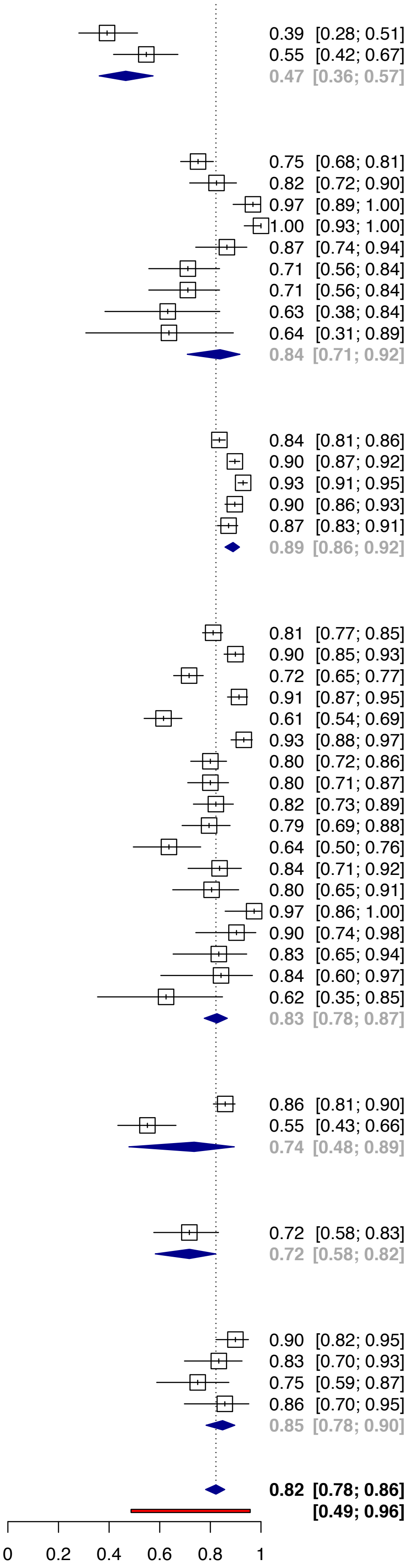

Study	N follow–up	N baseline	% Retention [95% CI]
**Eastern Mediterranean (Turkey)**			
Idilman 2008	29	74	0.39 [0.28; 0.51]
Omer 2010	35	64	0.55 [0.42; 0.67]
**Random effects model**	**64**	**138**	**0.47 [0.36; 0.57]**
Heterogeneity: $I^2 = 40\%$, $\tau^2 = < 0.1$, $\chi^2_1 = 3$ ($p = 0.07$)			
**Europe**			
Leuschner 2010	139	185	0.75 [0.68; 0.81]
Aithal 2008	61	74	0.82 [0.72; 0.90]
Ratziu 2008	61	63	0.97 [0.89; 1.00]
Georgescu 2009	54	54	1.00 [0.93; 1.00]
Armstrong 2016	45	52	0.87 [0.74; 0.94]
Dufour 2006	32	45	0.71 [0.56; 0.84]
McPherson 2017	32	45	0.71 [0.56; 0.84]
Sturm 2009	12	19	0.63 [0.38; 0.84]
Geier 2018	7	11	0.64 [0.31; 0.89]
**Random effects model**	**443**	**548**	**0.84 [0.71; 0.92]**
Heterogeneity: $I^2 = 89\%$, $\tau^2 = 1.1$, $\chi^2_8 = 18$ ($p = 0.02$)			
**Multiple regions**			
Younossi 2019	778	931	0.84 [0.81; 0.86]
Harrison 2020a– STELLAR 4	792	883	0.90 [0.87; 0.92]
Harrison 2020a – STELLAR 3	751	808	0.93 [0.91; 0.95]
Harrison 2020b	286	319	0.90 [0.86; 0.93]
Friedman 2018	252	289	0.87 [0.83; 0.91]
**Random effects model**	**2859**	**3230**	**0.89 [0.86; 0.92]**
Heterogeneity: $I^2 = 84\%$, $\tau^2 = < 0.1$, $\chi^2_4 = 39$ ($p < 0.01$)			
**North America**			
Harrison 2020c	326	402	0.81 [0.77; 0.85]
Sanyal 2010	222	247	0.90 [0.85; 0.93]
Sanyal 2014	174	243	0.72 [0.65; 0.77]
Neuschwander–Tetri 2015	200	219	0.91 [0.87; 0.95]
Lindor 2004	107	174	0.61 [0.54; 0.69]
Chalasani 2020	151	162	0.93 [0.88; 0.97]
Torres 2011	108	135	0.80 [0.72; 0.86]
Bril 2019	84	105	0.80 [0.71; 0.87]
Cusi 2016	83	101	0.82 [0.73; 0.89]
Navarro 2019	62	78	0.79 [0.69; 0.88]
Abdelmalek 2009	35	55	0.64 [0.50; 0.76]
Zein 2011	46	55	0.84 [0.71; 0.92]
Argo 2015	33	41	0.80 [0.65; 0.91]
Dasarathy 2015	36	37	0.97 [0.86; 1.00]
Promrat 2010	28	31	0.90 [0.74; 0.98]
Van Wagner 2011	25	30	0.83 [0.65; 0.94]
Shields 2009	16	19	0.84 [0.60; 0.97]
Nelson 2009	10	16	0.62 [0.35; 0.85]
**Random effects model**	**1746**	**2150**	**0.83 [0.78; 0.87]**
Heterogeneity: $I^2 = 85\%$, $\tau^2 = 0.4$, $\chi^2_{17} = 111$ ($p < 0.01$)			
**North America and Europe**			
Ratziu 2016	237	276	0.86 [0.81; 0.90]
Newsome 2020	43	78	0.55 [0.43; 0.66]
**Random effects model**	**280**	**354**	**0.74 [0.48; 0.89]**
Heterogeneity: $I^2 = 94\%$, $\tau^2 = 0.6$, $\chi^2_1 = 31$ ($p < 0.01$)			
**South America**			
Oliveira 2019	38	53	0.72 [0.58; 0.83]
**Random effects model**	**38**	**53**	**0.72 [0.58; 0.82]**
Heterogeneity: not applicable			
**Southeast Asia**			
Wah Kheong 2017	89	99	0.90 [0.82; 0.95]
Alam 2018	40	48	0.83 [0.70; 0.93]
Alam 2020	30	40	0.75 [0.59; 0.87]
Alam 2017	30	35	0.86 [0.70; 0.95]
**Random effects model**	**189**	**222**	**0.85 [0.78; 0.90]**
Heterogeneity: $I^2 = 24\%$, $\tau^2 = < 0.1$, $\chi^2_3 = 5$ ($p = 0.18$)			
**Random effects model**	**5619**	**6695**	**0.82 [0.78; 0.86]**
**Prediction interval**			**[0.49; 0.96]**
Heterogeneity: $I^2 = 92\%$, $\tau^2 = 0.6$, $\chi^2_{40} = 401$ ($p < 0.01$)			
Residual heterogeneity: $I^2 = 84\%$, $\chi^2_{34} = 207$ ($p < 0.01$)			

S8 Fig. Proportion of participants (95% CI) with a valid follow-up biopsy by trial duration.

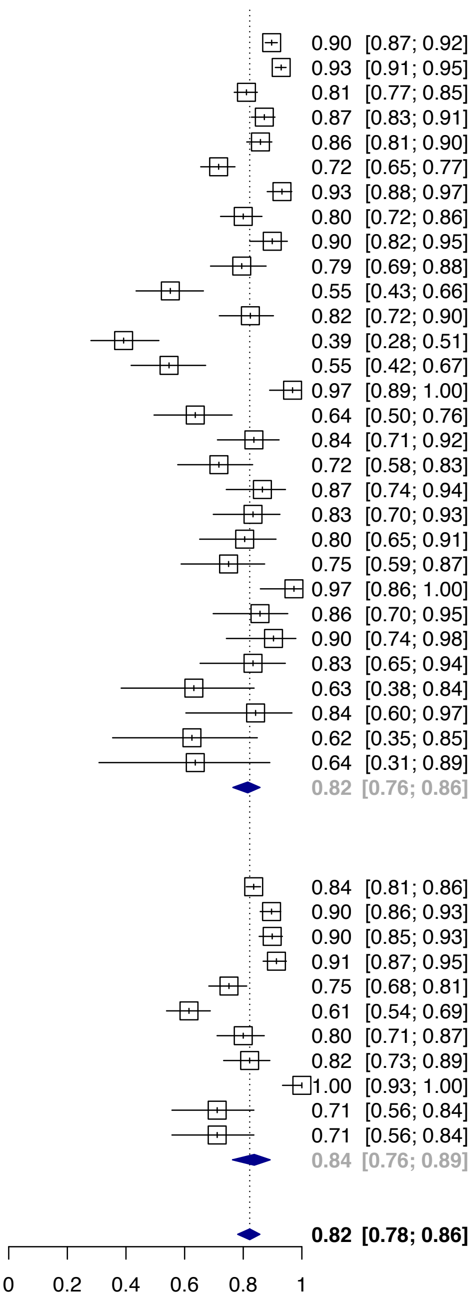

Study	N follow−up	N baseline	% Retention [95% CI]
**Length of follow−up = 1 year**			
Harrison 2020a− STELLAR 4	792	883	0.90 [0.87; 0.92]
Harrison 2020a − STELLAR 3	751	808	0.93 [0.91; 0.95]
Harrison 2020c	326	402	0.81 [0.77; 0.85]
Friedman 2018	252	289	0.87 [0.83; 0.91]
Ratziu 2016	237	276	0.86 [0.81; 0.90]
Sanyal 2014	174	243	0.72 [0.65; 0.77]
Chalasani 2020	151	162	0.93 [0.88; 0.97]
Torres 2011	108	135	0.80 [0.72; 0.86]
WahKheong 2017	89	99	0.90 [0.82; 0.95]
Navarro 2019	62	78	0.79 [0.69; 0.88]
Newsome 2020	43	78	0.55 [0.43; 0.66]
Aithal 2008	61	74	0.82 [0.72; 0.90]
Idilman 2008	29	74	0.39 [0.28; 0.51]
Omer 2010	35	64	0.55 [0.42; 0.67]
Ratziu 2008	61	63	0.97 [0.89; 1.00]
Abdelmalek 2009	35	55	0.64 [0.50; 0.76]
Zein 2011	46	55	0.84 [0.71; 0.92]
Oliveira 2019	38	53	0.72 [0.58; 0.83]
Armstrong 2016	45	52	0.87 [0.74; 0.94]
Alam 2018	40	48	0.83 [0.70; 0.93]
Argo 2015	33	41	0.80 [0.65; 0.91]
Alam 2020	30	40	0.75 [0.59; 0.87]
Dasarathy 2015	36	37	0.97 [0.86; 1.00]
Alam 2017	30	35	0.86 [0.70; 0.95]
Promrat 2010	28	31	0.90 [0.74; 0.98]
VanWagner 2011	25	30	0.83 [0.65; 0.94]
Sturm 2009	12	19	0.63 [0.38; 0.84]
Shields 2009	16	19	0.84 [0.60; 0.97]
Nelson 2009	10	16	0.62 [0.35; 0.85]
Geier 2018	7	11	0.64 [0.31; 0.89]
**Random effects model**	**3602**	**4270**	**0.82 [0.76; 0.86]**
Heterogeneity: $I^2 = 91\%$, $\tau^2 = 0.6$, $\chi^2_{29} = 306$ ($p < 0.01$)			
**Length of follow−up = More than 1 year**			
Younossi 2019	778	931	0.84 [0.81; 0.86]
Harrison 2020b	286	319	0.90 [0.86; 0.93]
Sanyal 2010	222	247	0.90 [0.85; 0.93]
Neuschwander−Tetri 2015	200	219	0.91 [0.87; 0.95]
Leuschner 2010	139	185	0.75 [0.68; 0.81]
Lindor 2004	107	174	0.61 [0.54; 0.69]
Bril 2019	84	105	0.80 [0.71; 0.87]
Cusi 2016	83	101	0.82 [0.73; 0.89]
Georgescu 2009	54	54	1.00 [0.93; 1.00]
Dufour 2006	32	45	0.71 [0.56; 0.84]
McPherson 2017	32	45	0.71 [0.56; 0.84]
**Random effects model**	**2017**	**2425**	**0.84 [0.76; 0.89]**
Heterogeneity: $I^2 = 93\%$, $\tau^2 = 0.5$, $\chi^2_{10} = 94$ ($p < 0.01$)			
**Random effects model**	**5619**	**6695**	**0.82 [0.78; 0.86]**
Heterogeneity: $I^2 = 92\%$, $\tau^2 = 0.6$, $\chi^2_{40} = 401$ ($p < 0.01$)			
Residual heterogeneity: $I^2 = 90\%$, $\chi^2_{39} = 400$ ($p < 0.01$)			

Test for subgroup differences (random effects model): p-value=0.59.
Following feedback from peer-reviewers, we also conducted a meta-regression by trial duration. This confirmed the lack of evidence of a difference in the proportion of follow-up biopsies in trials with duration more than 1 year compared with less than 1 year [b = 0.16 (95% CI: -0.41 to 0.73, p=0.58)].

S9 Fig. Comparison of proportions of participants with valid follow-up biopsies in studies with an additional follow-up biopsy

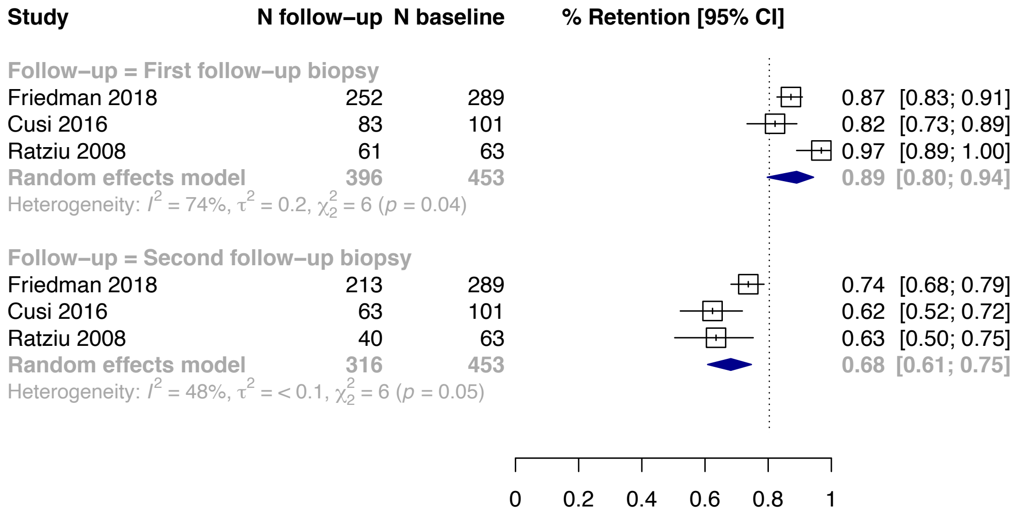

S10 Fig. Odds ratio (95% CI) of the proportion of participants with a valid follow-up biopsy between trial arms in placebo-controlled trials.

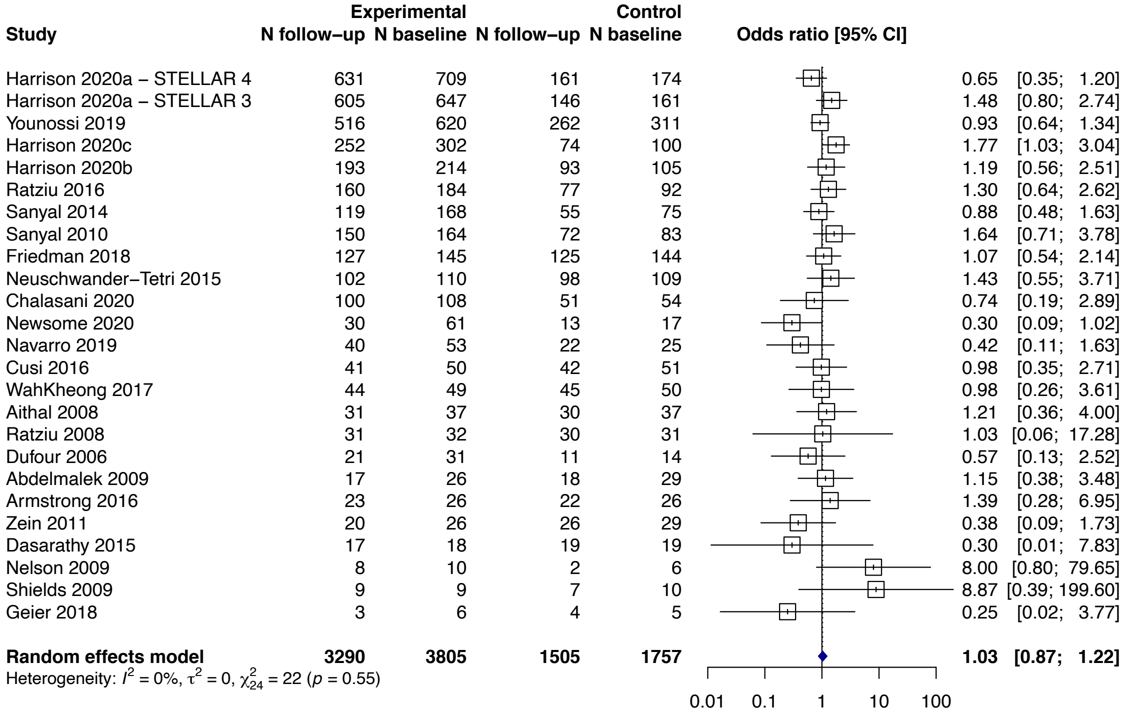

S11 Fig. Odds ratio (95% CI) of the proportion of participants with a valid follow-up biopsy between trial arms in the open-label two-arm trials. PEoB: Potential expectation of benefit.

Study	Higher PEoB N follow−up	Higher PEoB N baseline	Lower PEoB N follow−up	Lower PEoB N baseline	Odds ratio [95% CI]
Torres 2011	77	94	31	41	1.46 [0.60; 3.54]
Idilman 2008	21	49	8	25	1.59 [0.58; 4.39]
Oliveira 2019	20	26	18	27	1.67 [0.50; 5.61]
Alam 2017	20	25	10	10	0.18 [0.01; 3.53]
Alam 2018	20	24	20	24	1.00 [0.22; 4.56]
Omer 2010	12	22	23	42	0.99 [0.35; 2.79]
Promrat 2010	18	21	10	10	0.25 [0.01; 5.36]
Alam 2020	16	20	14	20	1.71 [0.40; 7.34]
Sturm 2009	5	10	7	9	0.29 [0.04; 2.11]
**Random effects model**	**209**	**291**	**141**	**208**	**1.11 [0.72; 1.70]**

Heterogeneity: $I^2 = 0\%$, $\tau^2 = 0$, $\chi^2_8 = 3$ ($p = 0.93$)

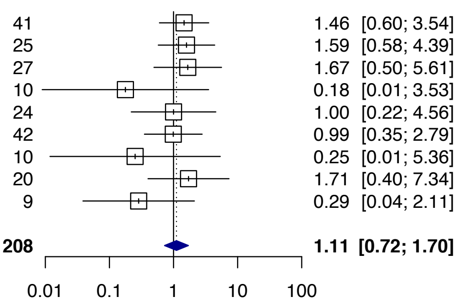

S12 Fig. Funnel plot

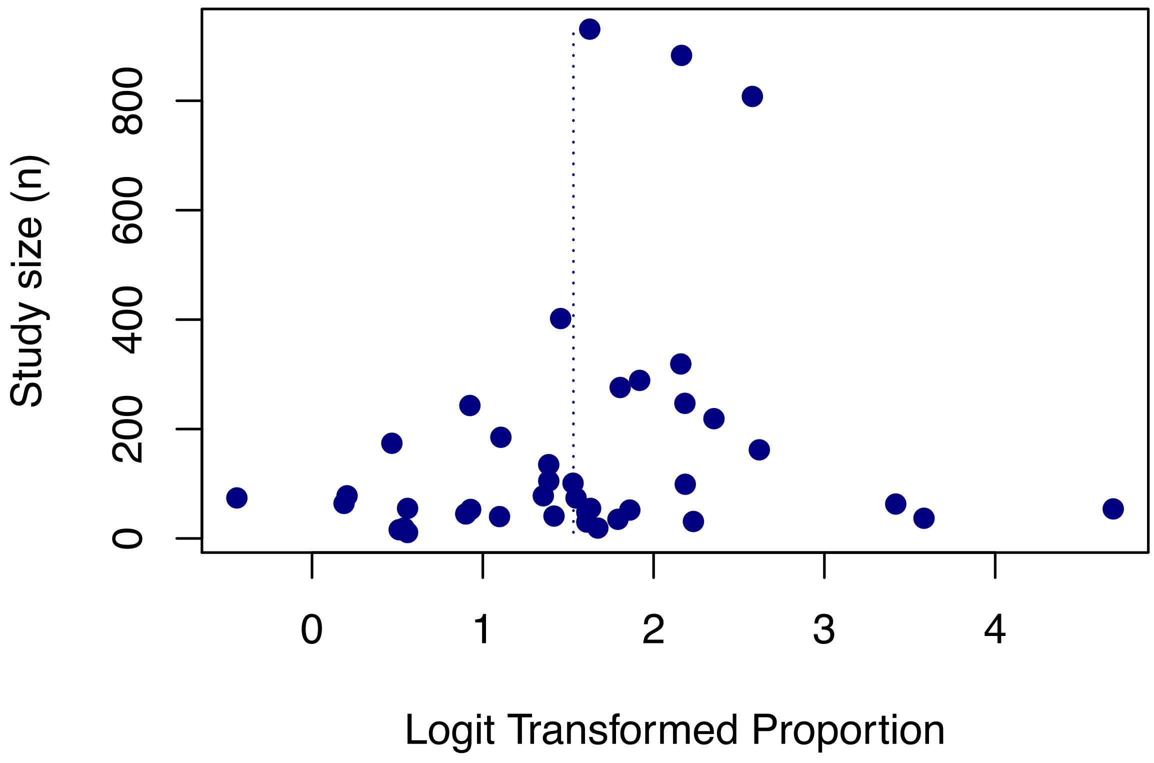

S2 Table. Quality assessment: risk of bias due to blinding of participants and personnel

Study	Risk of bias due to blinding
Alam 2017	High risk
Alam 2018	High risk
Alam 2020	High risk
Idilman 2008	High risk
Oliveira 2019	High risk
Omer 2010	High risk
Sturm 2009	High risk
Torres 2011	High risk
Abdelmalek 2009	Low risk
Aithal 2008	Low risk
Argo 2015	Low risk
Armstrong 2016	Low risk
Bril 2019	Low risk
Chalasani 2020	Low risk
Cusi 2016	Low risk
Dasarathy 2015	Low risk
Dufour 2006	Low risk
Friedman 2018	Low risk
Geier 2018	Low risk
Georgescu 2009	Low risk
Harrison 2020a - STELLAR 3	Low risk
Harrison 2020a- STELLAR 4	Low risk
Harrison 2020b	Low risk
Harrison 2020c	Low risk
Leuschner 2010	Low risk
Lindor 2004	Low risk
McPherson 2017	Low risk
Navarro 2019	Low risk
Nelson 2009	Low risk
Neuschwander-Tetri 2015	Low risk
Newsome 2020	Low risk
Ratziu 2008	Low risk
Ratziu 2016	Low risk
Sanyal 2010	Low risk
Sanyal 2014	Low risk
Shields 2009	Low risk
Van Wagner 2011	Low risk
Wah Kheong 2017	Low risk
Younossi 2019	Low risk
Zein 2011	Low risk
Promrat 2010	Not applicable

Note: We decided a priori not to assess risk of bias due to randomization and allocation, as we did not consider that they could meaningfully bias the primary outcome which itself is part of the tool (i.e. as attrition bias).

Search strategy

Medline search

# ▲	Searches
1	Non-alcoholic Fatty Liver Disease/
2	*Fatty Liver/
3	((nonalcoholic or non-alcoholic) adj5 (fatty liver or steatohepatitis)).ti,ab.
4	(fatty liver or steatohepatitis).ti.
5	(nafld or nash).ti,ab.
6	1 or 2 or 3 or 4 or 5
7	biopsy/ or exp biopsy, needle/ or exp image-guided biopsy/
8	Liver/cy, pa or Fatty Liver/pa
9	(biopsy or biopsies).ti,ab.
10	((liver or hepatic) adj2 (patholog* or histolog* or cytolog*)).ti,ab.
11	7 or 8 or 9 or 10
12	6 and 11
13	randomized controlled trial.pt.
14	controlled clinical trial.pt.
15	randomized.ab.
16	placebo.ab.
17	clinical trials as topic.sh.
18	randomly.ab.
19	trial.ti.
20	13 or 14 or 15 or 16 or 17 or 18 or 19
21	exp animals/ not humans.sh.
22	20 not 21
23	12 and 22

Embase search

# ▲	Searches
1	exp *nonalcoholic fatty liver/
2	*Fatty Liver/
3	((nonalcoholic or non-alcoholic) adj5 (fatty liver or steatohepatitis)).ti,ab.
4	(fatty liver or steatohepatitis).ti.
5	(nafld or nash).ti,ab.
6	1 or 2 or 3 or 4 or 5
7	liver biopsy/ or biopsy/
8	(liver/ or fatty liver/) and (cytology/ or histology/)
9	(biopsy or biopsies).ti,ab.
10	((liver or hepatic) adj2 (patholog* or histolog* or cytolog*)).ti,ab.
11	7 or 8 or 9 or 10
12	6 and 11
13	randomized controlled trial/
14	single blind procedure/ or double blind procedure/
15	crossover procedure/
16	random*.tw.
17	(random or ((singl* or doubl*) adj (blind* or mask*)) or crossover or cross over or factorial* or latin square or assign* or allocat* or volunteer*).ti,ab.
18	13 or 14 or 15 or 16 or 17
19	(exp animals/ or nonhuman/) not

human/ 20 18 not 19

21	12 and 20

References of included studies

[1] Chalasani N, Abdelmalek MF, Garcia-Tsao G*, et al.* Effects of Belapectin, an Inhibitor of Galectin-3, in Patients With Nonalcoholic Steatohepatitis With Cirrhosis and Portal Hypertension. *Gastroenterology*. 2020; **158**: 1334-45.e5.

[2] Abdelmalek MF, Sanderson SO, Angulo P*, et al.* Betaine for nonalcoholic fatty liver disease: results of a randomized placebo-controlled trial. *Hepatology*. 2009; **50**: 1818-26.

[3] Friedman SL, Ratziu V, Harrison SA*, et al.* A randomized, placebo-controlled trial of cenicriviroc for treatment of nonalcoholic steatohepatitis with fibrosis. *Hepatology*. 2018; **67**: 1754-67.

[4] Ratziu V, Sanyal A, Harrison SA*, et al.* Cenicriviroc Treatment for Adults with Nonalcoholic Steatohepatitis and Fibrosis: Final Analysis of the Phase 2b CENTAUR Study. *Hepatology*. 2020; **13**: 13.

[5] Ratziu V, Harrison SA, Francque S*, et al.* Elafibranor, an Agonist of the Peroxisome Proliferator-Activated Receptor-alpha and -delta, Induces Resolution of Nonalcoholic Steatohepatitis Without Fibrosis Worsening. *Gastroenterology*. 2016; **150**: 1147-59.e5.

[6] Harrison SA, Goodman Z, Jabbar A*, et al.* A randomized, placebo-controlled trial of emricasan in patients with NASH and F1-F3 fibrosis. *Journal of Hepatology*. 2020; **72**: 816-27.

[7] Sanyal AJ, Abdelmalek MF, Suzuki A, Cummings OW, Chojkier M, Group E-AS. No significant effects of ethyl-eicosapentanoic acid on histologic features of nonalcoholic steatohepatitis in a phase 2 trial. *Gastroenterology*. 2014; **147**: 377-84.e1.

[8] Idilman R, Mizrak D, Corapcioglu D*, et al.* Clinical trial: insulin-sensitizing agents may reduce consequences of insulin resistance in individuals with non-alcoholic steatohepatitis. *Alimentary Pharmacology & Therapeutics*. 2008; **28**: 200-8.

[9] Armstrong MJ, Gaunt P, Aithal GP*, et al.* Liraglutide safety and efficacy in patients with non-alcoholic steatohepatitis (LEAN): a multicentre, double-blind, randomised, placebo-controlled phase 2 study. *Lancet*. 2016; **387**: 679-90.

[10] McPherson S, Wilkinson N, Tiniakos D*, et al.* A randomised controlled trial of losartan as an anti-fibrotic agent in non-alcoholic steatohepatitis. *PLoS ONE [Electronic Resource]*. 2017; **12**: e0175717.

[11] Sturm N, Bronowicki JP, Maynard-Muet M*, et al.* Metformin plus pentoxifylline versus prescriptive diet in non-alcoholic steatohepatitis (NASH): a randomized controlled pilot trial. *Gastroenterologie Clinique et Biologique*. 2009; **33**: 984-6.

[12] Shields WW, Thompson KE, Grice GA, Harrison SA, Coyle WJ. The Effect of Metformin and Standard Therapy versus Standard Therapy alone in Nondiabetic Patients with Insulin Resistance and Nonalcoholic Steatohepatitis (NASH): A Pilot Trial. *Therapeutic Advances in Gastroenterology*. 2009; **2**: 157-63.

[13] Omer Z, Cetinkalp S, Akyildiz M*, et al.* Efficacy of insulin-sensitizing agents in nonalcoholic fatty liver disease. *European Journal of Gastroenterology & Hepatology*. 2010; **22**: 18-23.

[14] Harrison SA, Alkhouri N, Davison BA*, et al.* Insulin sensitizer MSDC-0602K in non-alcoholic steatohepatitis: A randomized, double-blind, placebo-controlled phase IIb study. *Journal of Hepatology*. 2020; **72**: 613-26.

[15] Dasarathy S, Dasarathy J, Khiyami A*, et al.* Double-blind randomized placebo-controlled clinical trial of omega 3 fatty acids for the treatment of diabetic patients with nonalcoholic steatohepatitis. *Journal of Clinical Gastroenterology*. 2015; **49**: 137-44.

[16] Argo CK, Patrie JT, Lackner C*, et al.* Effects of n-3 fish oil on metabolic and histological parameters in NASH: a double-blind, randomized, placebo-controlled trial. *Journal of Hepatology*. 2015; **62**: 190-7.
[17] Oliveira CP, Cotrim HP, Stefano JT, Siqueira ACG, Salgado ALA, Parise ER. N-Acetylcysteine and/or Ursodeoxycholic Acid Associated with Metformin in Non-Alcoholic Steatohepatitis: An Open-Label Multicenter Randomized Controlled Trial. *Arquivos de Gastroenterologia*. 2019; **56**: 184-90.
[18] Younossi ZM, Ratziu V, Loomba R*, et al.* Obeticholic acid for the treatment of non-alcoholic steatohepatitis: interim analysis from a multicentre, randomised, placebo-controlled phase 3 trial. *Lancet*. 2019; **394**: 2184-96.
[19] Neuschwander-Tetri BA, Loomba R, Sanyal AJ*, et al.* Farnesoid X nuclear receptor ligand obeticholic acid for non-cirrhotic, non-alcoholic steatohepatitis (FLINT): a multicentre, randomised, placebo-controlled trial. *Lancet*. 2015; **385**: 956-65.
[20] Van Wagner LB, Koppe SW, Brunt EM*, et al.* Pentoxifylline for the treatment of non-alcoholic steatohepatitis: a randomized controlled trial. *Annals of Hepatology*. 2011; **10**: 277-86.
[21] Zein CO, Yerian LM, Gogate P*, et al.* Pentoxifylline improves nonalcoholic steatohepatitis: a randomized placebo-controlled trial. *Hepatology*. 2011; **54**: 1610-9.
[22] Alam S, Nazmul Hasan S, Mustafa G, Alam M, Kamal M, Ahmad N. Effect of Pentoxifylline on Histological Activity and Fibrosis of Nonalcoholic Steatohepatitis Patients: A One Year Randomized Control Trial. *Journal of Translational Internal Medicine*. 2017; **5**: 155-63.
[23] Cusi K, Orsak B, Bril F*, et al.* Long-Term Pioglitazone Treatment for Patients With Nonalcoholic Steatohepatitis and Prediabetes or Type 2 Diabetes Mellitus: A Randomized Trial. *Annals of Internal Medicine*. 2016; **165**: 305-15.
[24] Aithal GP, Thomas JA, Kaye PV*, et al.* Randomized, placebo-controlled trial of pioglitazone in nondiabetic subjects with nonalcoholic steatohepatitis. *Gastroenterology*. 2008; **135**: 1176-84.
[25] Ratziu V, Giral P, Jacqueminet S*, et al.* Rosiglitazone for nonalcoholic steatohepatitis: one-year results of the randomized placebo-controlled Fatty Liver Improvement with Rosiglitazone Therapy (FLIRT) Trial. *Gastroenterology*. 2008; **135**: 100-10.
[26] Torres DM, Jones FJ, Shaw JC, Williams CD, Ward JA, Harrison SA. Rosiglitazone versus rosiglitazone and metformin versus rosiglitazone and losartan in the treatment of nonalcoholic steatohepatitis in humans: a 12-month randomized, prospective, open- label trial. *Hepatology*. 2011; **54**: 1631-9.
[27] Harrison SA, Wong VW, Okanoue T*, et al.* Selonsertib for patients with bridging fibrosis or compensated cirrhosis due to NASH: Results from randomized phase III STELLAR trials. *Journal of Hepatology*. 2020; **06**: 06.
[28] Navarro VJ, Belle SH, D'Amato M*, et al.* Silymarin in non-cirrhotics with non-alcoholic steatohepatitis: A randomized, double-blind, placebo controlled trial. *PLoS ONE [Electronic Resource]*. 2019; **14**: e0221683.
[29] Wah Kheong C, Nik Mustapha NR, Mahadeva S. A Randomized Trial of Silymarin for the Treatment of Nonalcoholic Steatohepatitis. *Clinical Gastroenterology & Hepatology*. 2017; **15**: 1940-9.e8.
[30] Nelson A, Torres DM, Morgan AE, Fincke C, Harrison SA. A pilot study using simvastatin in the treatment of nonalcoholic steatohepatitis: A randomized placebo-controlled trial. *Journal of Clinical Gastroenterology*. 2009; **43**: 990-4.

[31] Alam S, Ghosh J, Mustafa G, Kamal M, Ahmad N. Effect of sitagliptin on hepatic histological activity and fibrosis of nonalcoholic steatohepatitis patients: a 1-year randomized control trial. *Hepatic Medicine Evidence and Research*. 2018; **10**: 23-31.
[32] Leuschner UF, Lindenthal B, Herrmann G*, et al.* High-dose ursodeoxycholic acid therapy for nonalcoholic steatohepatitis: a double-blind, randomized, placebo-controlled trial. *Hepatology*. 2010; **52**: 472-9.
[33] Lindor KD, Kowdley KV, Heathcote EJ*, et al.* Ursodeoxycholic acid for treatment of nonalcoholic steatohepatitis: results of a randomized trial. *Hepatology*. 2004; **39**: 770-8.
[34] Dufour JF, Oneta CM, Gonvers JJ*, et al.* Randomized placebo-controlled trial of ursodeoxycholic acid with vitamin e in nonalcoholic steatohepatitis. *Clinical Gastroenterology & Hepatology*. 2006; **4**: 1537-43.
[35] Georgescu EF, Ionescu R, Niculescu M, Mogoanta L, Vancica L. Angiotensin-receptor blockers as therapy for mild-to-moderate hypertension-associated non-alcoholic steatohepatitis. *World Journal of Gastroenterology*. 2009; **15**: 942-54.
[36] Geier A, Eichinger M, Stirnimann G*, et al.* Treatment of non-alcoholic steatohepatitis patients with vitamin D: a double-blinded, randomized, placebo-controlled pilot study. *Scandinavian Journal of Gastroenterology*. 2018; **53**: 1114-20.
[37] Bril F, Biernacki DM, Kalavalapalli S*, et al.* Role of Vitamin E for Nonalcoholic Steatohepatitis in Patients With Type 2 Diabetes: A Randomized Controlled Trial. *Diabetes Care*. 2019; **42**: 1481-8.
[38] Sanyal AJ, Chalasani N, Kowdley KV*, et al.* Pioglitazone, vitamin E, or placebo for nonalcoholic steatohepatitis. *New England Journal of Medicine*. 2010; **362**: 1675-85.
[39] Alam S, Abrar M, Islam S*, et al.* Effect of telmisartan and vitamin E on liver histopathology with non-alcoholic steatohepatitis: A randomized, open-label, noninferiority trial. *JGH Open*. 2020.
[40] Newsome PN, Palmer M, Freilich B*, et al.* Volixibat in adults with non-alcoholic steatohepatitis: 24-week interim analysis from a randomized, phase II study. *Journal of Hepatology*. 2020; **28**: 28.
[41] Promrat K, Kleiner DE, Niemeier HM*, et al.* Randomized controlled trial testing the effects of weight loss on nonalcoholic steatohepatitis. *Hepatology*. 2010; **51**: 121-9.
