# Supplementary material for: What proportion of people have a follow-up biopsy in randomized trials of treatments for non-alcoholic steatohepatitis?: A systematic review and meta-analysis
Source: PLoS One. 2021 Apr 21;16(4):e0250385. doi: 10.1371/journal.pone.0250385 (PMC8059856; doi:10.1371/journal.pone.0250385)
Supplement: Attachment — Submitted filename: Response to reviewers.docx [file pone.0250385.s003.docx]

1. Please ensure that your manuscript meets PLOS ONE's style requirements, including those for file naming. The PLOS ONE style templates can be found at

<https://journals.plos.org/plosone/s/file?id=wjVg/PLOSOne_formatting_sample_main_body.pdf> and

[https://journals.plos.org/plosone/s/file?id=ba62/PLOSOne_formatting_sample_title_authors_affiliations.pdf](about:blank)

Response: Thank you, we have edited the manuscript to reflect the above style templates.

2. Please described the data extraction methods in more details. We would expect to see reporting of the specific information extracted from the manuscripts.

Response: We have now added information on the specific data that were extracted. These data match the pre-specified data extraction plan in the prospectively published protocol.

Lines 99-103 (in the clean version of the manuscript): Following this, two researchers independently extracted the following data: baseline characteristics (age, sex, NAFLD activity score, fibrosis stage), nature of intervention, number of people with a biopsy at baseline and follow-up, reasons for lack of follow-up biopsy, biopsy-related adverse events, length and timings of follow-up, and reimbursement for study participation.

Reviewers' comments:

Reviewer's Responses to Questions

Reviewer #1: In this paper, Koutoukidis et al. assessed quantify the proportion of participants with a valid follow-up biopsy. They found that the proportion of participants with a valid follow-up biopsy was 82%. There was no evidence of a difference by location, trial length, or by allocated treatment group. Reasons for missing follow-up biopsies were, in ranked order, related to participants, medical factors, protocol, trial conduct, and other/unclear. The present study deals with an important topic and may provide important information for the clinical trial for NAFLD/NASH. This meta-analysis will be improved by revising following issues.

Response: Thank you for your positive comment.

Major comments
#1. As the results showed, repeated biopsy could not be performed in approximately 20% of enrolled subjects even in clinical trials. These findings indicated the importance of non-invasive tests as a main outcome. Recently, magnetic resonance elastography (MRE) is reported have high diagnostic accuracy (PMID: 29908362, PMID: 31556124, PMID: 30291868, PMID: 30014566). The authors should discuss the importance of non-invasive tests in clinical trials by referring the above reports.

Response: Thank you for this point and the suggested literature. We have added this in our discussion.

Lines 250-252: Non-invasive biomarkers, such as magnetic resonance imaging and elastography, are emerging as potential alternatives to the liver biopsy that will minimise such adverse events.

#2. Recently, a new definition for metabolic dysfunction-associated fatty liver disease (MAFLD) has been proposed by an international expert consensus panel (PMID: 32278004). In MAFLD criteria, liver biopsy is not required for the diagnosis and MAFLD is associated with hepatic fibrosis than NAFLD (PMID: 32478487, PMID: 32997882) indicating that non-invasive tests/metabolic dysfunction can be main outcome in the MAFLD definition. The authors should discuss the future perspective in MAFLD ear.

Response: Thank you for this point and we agree this is an interesting and promising proposal. We have not changed our terminology here because NAFLD is the dominant term and these proposals are not yet widely approved and used. However, we have acknowledged the issue in the discussion as follows:

Lines 252-254: The new proposed definition of metabolic dysfunction-associated fatty liver disease might reinforce the use of such biomarkers for diagnosis and as trial endpoints, although the optimal terminology remains to be determined.

#3. Meta-regression analysis is better to be added in this study.

Response: Thank you for this suggestion. We have now run meta-regressions for the 2 main subgroup analyses (by location and trial duration). The results and interpretation did not change.

Legend of Fig S6: Test for subgroup differences (random effects model): p-value=0.11. Following feedback from peer-reviewers, we also conducted a meta-regression by location. This confirmed the lack of evidence of a difference in the proportion of follow-up biopsies in middle-income compared with high-income countries [b = -0.60 (95% CI: -1.26 to 0.05, p=0.07)].

Legend of Fig S8: Test for subgroup differences (random effects model): p-value=0.59. Following feedback from peer-reviewers, we also conducted a meta-regression by trial duration. This confirmed the lack of evidence of a difference in the proportion of follow-up biopsies in trials with duration more than 1 year compared with less than 1 year [b = 0.16 (95% CI: -0.41 to 0.73, p=0.58)].

Minor comments
#1. In conclusion (line 58), the authors conclude that “…is on average 82%, with around 1 in 10 participants declining..”. It should be “approximately 2 in 10 participants declining..”

Response: Thank you for this comment. We would like to clarify that the “1 in 10 participants declining” is based on our analysis of participants specifically declining a second liver biopsy. The 82% estimate encompasses both that some participants decline as well as all other factors that a second biopsy was not feasible (e.g. insufficient sampling). Therefore, we did not make changes to the conclusion.

#2. Quality assessment should be performed according to the criteria formulated by the Cochran Effective Practice and Organization of Care (EPOC) group. The authors should assess the followings: random sequence generation, allocation concealment, blinding of participants and researchers, blinding of outcome assessment, incomplete outcome data, selective reporting, other bias.

Response: Thank you for this point and we agree that these are criteria typically seen in meta-analyses (and ones we have used in our other previous meta-analyses). However, as stated in the methods, we had pre-specified in the published protocol of the review that we would only assess bias due to blinding and we have reported this in the manuscript.

Randomisation and allocation:

The rationale for not assessing bias due to random sequence generation and due to allocation concealment was that such biases are unlikely to meaningfully affect our primary outcomes (i.e. percentage of participants with a valid biopsy at follow-up and the percentage of participants declining a follow-up biopsy). These biases affect differences between arms, but our outcome derives from both arms considered together. This was confirmed by our sensitivity analysis that found no evidence of a difference in the proportion of follow-up biopsies between groups among both placebo-controlled and open-label trials (Figs S10-S11). Such potential biases were also unlikely to affect biopsy-related adverse events.

Incomplete outcome data:

By definition of one of our primary outcomes (percentage of participants with a valid biopsy at follow-up), we assessed bias due to incomplete outcome data, so we did not feel that it was appropriate to also mention it as part of the bias assessment.

Selective reporting:

Selective reporting cannot bias our primary outcome, the proportion of follow-up biopsies, since all studies reported on it. Selective reporting could affect our estimates of adverse effects, although we doubt this is a big effect. It is common practice to report only on the adverse events that occur (rather than reporting that specific adverse events were absent), so it seems likely that not reporting adverse events implies no adverse events occurred. However, we accounted for this potential bias by estimating the occurrence of adverse events from biopsies using data from only those studies specifically reporting adverse events and, thus, the decision to report biopsy-related events is unrelated to their occurrence in these trials.

Other:

We had considered other potential biases at the design stage, but we did not identify any specific ones and, therefore, this type of bias was not applicable here.

Reviewer #2: This is a systematic review and meta-analysis regarding what proportion of participants have a follow-up biopsy in randomized trials of treatments for non-alcoholic steatohepatitis (NASH). There are forty-one randomized clinical trials in NASH with a total of 6,695 participants included in this systematic review. The results demonstrated that the proportion of participants with a valid follow-up biopsy was on average 82% (78-86%), but with unexplained heterogeneity between trials. The reasons for missing follow-up biopsies were related to participants, medical factors, protocol, trial conduct, and other/unclear reasons. Biopsy-related serious adverse events occurred in 16 per 1,000 participants.
Liver biopsies are invasive and have potential risks including pain, bleeding, bile leak and even death for patients. Participants particularly in placebo control groups who did not receive therapy may hesitate to undertake a follow-up liver biopsy.
It is important to know that whether participants are willing to undergo a follow-up liver biopsy are also affected by geographic and cultural differences. However, these factors are not take into consideration in this review.

Response: Thank you for this point. In our original manuscript, we had aimed to address this point with subgroup analysis of the primary outcome among high-income vs. low-income countries. We have now added an analysis by geographical region. This confirmed the results of our original analysis showing lack of clear evidence of geographical and cultural differences.

Lines 251-256: There was no evidence that the location (high-income vs. middle-income counties) influenced the proportion of valid follow-up biopsies (S6 Fig). In the analysis by geographical region, there was some limited evidence that this proportion was lower in two small studies conducted in Eastern Mediterranean but not in any other region (S7 Fig).
